# Characterization of Bacterial Communities Associated with the Tyrian Purple Producing Gland in a Marine Gastropod

**DOI:** 10.1371/journal.pone.0140725

**Published:** 2015-10-21

**Authors:** Ajit Kumar Ngangbam, Abdul Baten, Daniel L. E. Waters, Steve Whalan, Kirsten Benkendorff

**Affiliations:** 1 Marine Ecology Research Centre, School of Environment, Science and Engineering, Southern Cross University, Lismore, NSW 2480, Australia; 2 Southern Cross Plant Science, Southern Cross University, Lismore, NSW 2480, Australia; Catalan Institute for Water Research (ICRA), SPAIN

## Abstract

*Dicathais orbita* is a marine mollusc recognised for the production of anticancer compounds that are precursors to Tyrian purple. This study aimed to assess the diversity and identity of bacteria associated with the Tyrian purple producing hypobranchial gland, in comparison with foot tissue, using a high-throughput sequencing approach. Taxonomic and phylogenetic analysis of variable region V1-V3 of 16S rRNA bacterial gene amplicons in QIIME and MEGAN were carried out. This analysis revealed a highly diverse bacterial assemblage associated with the hypobranchial gland and foot tissues of *D*. *orbita*. The dominant bacterial phylum in the 16S rRNA bacterial profiling data set was *Proteobacteria* followed by *Bacteroidetes*, *Tenericutes* and *Spirochaetes*. In comparison to the foot, the hypobranchial gland had significantly lower bacterial diversity and a different community composition, based on taxonomic assignment at the genus level. A higher abundance of indole producing *Vibrio* spp. and the presence of bacteria with brominating capabilities in the hypobranchial gland suggest bacteria have a potential role in biosynthesis of Tyrian purple in *D*. *orbita*.

## Introduction

Tyrian purple is a dye of historical and religious importance [1, 2] and its indole precursors are reported to have potential anticancer and antimicrobial properties [3–8]. Muricid molluscs are the only natural source of Tyrian purple [2], which is formed as a secondary metabolite from indoxyl sulfate precursors stored in the hypobranchial gland [4, 9]. The main pigment in Tyrian purple (6, 6’ dibromoindigo) was the first marine natural product to be structurally elucidated [10], however, even a century later, little information is available on its biosynthesis or the potential role of endosymbiotic bacteria in its production [4]. 6, 6’ Dibromoindigo is the brominated derivative of the blue dye indigo, produced by plants [11, 12] and a range of bacteria [13–15]. The formation of halogenated marine natural products (mostly containing chlorine and bromine), requires enzymes, such as halogenases and haloperoxidases [16]. Bromoperoxidases are believed to be involved in the bromination of indoxyl sulfate precursors, resulting in Tyrian purple biosynthesis in muricid molluscs [17, 18]. Several marine bacteria such as *Psychrobacter* sp., *Vibrio* sp., *Pseudomonas* sp. and *Streptomyces* sp. produce halogenases [19–23], while *Pseudomonas* sp. [24–26], *Streptomyces* sp. [27–30] and *Synechococcus* sp. [31] are known to produce bromoperoxidases.

Bacterial profiling using pyrosequencing is an efficient approach for identifying the diversity of endo-symbiotic bacteria and their interactions within marine invertebrates. For instance, metagenome analysis has revealed the remarkable diversity of bacterial symbionts in sponges [32, 33] and the presence of biosynthetic genes in sponge microbial symbionts [34, 35]. Other studies have highlighted the host–symbiont biochemical interactions between *Proteobacterium* sp. and the deep sea tube worm, *Riftia* sp. [36–38]. Thus, targeted metagenomic studies can elucidate a range of species associations and functional relationships.

The Australian muricid, *Dicathais orbita*, provides a useful model for studying Tyrian purple, and the biosynthesis of anticancer brominated indoles more generally [4]. In preliminary studies, three indole producing bacteria were cultured from the Tyrian purple producing hypobranchial glands of *D*. *orbita*. However, these studies relied on traditional culture methods and yielded a relatively low number of bacteria, with just 16 distinct strains isolated from all tissues, only three of which were from the Tyrian purple producing gland [39]. Culturing most marine bacteria is difficult, with only an estimated 0.001–0.1% of marine microbes being successfully cultured [40]. Therefore, the aim of this study was to assess the diversity of bacteria associated with the Tyrian purple producing hypobranchial gland using high-throughput sequencing (454 GS FLX Titanium) of the variable region V1-V3 of the 16S rRNA bacterial gene. Comparison of these sequences with equivalent sequences isolated from foot tissue will contribute to the identification of bacteria specifically associated with, or more abundant in, the Tyrian purple producing hypobranchial gland. A further aim was to identify bacteria with potential to produce indoles and brominated compounds, based on their taxonomic affiliation.

## Materials and Methods

### Sample collection and maintenance

Adult specimens of *D*. *orbita* were collected under permit number F89/1171-6.0 and P10/0069-1.0 issued by Department of Primary Industries, (NSW) Australia. Six live snails were collected from the intertidal rocky reefs of Flat Rock, Ballina (28°84′ S and 153°60′ E), NSW, Australia, during low tides in April and July 2014. Snails were held in aerated seawater tanks for a maximum of 24 hours before processing.

### Snail dissection and total DNA extraction

The hard shell of *D*. *orbita* was removed by applying pressure between the primary body whorl and spire using a bench vice [41]. The hypobranchial glands and the foot were carefully rinsed by pipetting with sterile sea water to remove any sediment before dissecting. Total genomic DNA from triplicate female and male hypobranchial glands, as well as the female and male foot, was extracted using the QIAmp DNA mini kit (Qiagen) following the manufacturer’s instructions. DNA quality and concentrations were determined with agarose gel electrophoresis and a NanoDrop 2000 Spectrophotometer (Thermo Scientific) and then stored at –20°C pending analysis. Only those samples that passed quality control checks were used in the 16s rDNA bacterial profiling libraries, so in total only duplicate samples were obtained for each gender and tissue combination.

### Roche GS- FLX amplicon sequencing

Bacterial diversity of the biosynthetic organ (hypobranchial glands) and non-biosynthetic tissues (foot) of *D*. *orbita* were characterised by high-throughput sequencing (454 GS FLX amplicon sequencing) [42] using the primer pair of 27F/519R that targeted the variable region V1-V3 of 16S rRNA bacterial gene [43, 44]. DNA samples were shipped to Macrogen Inc, South Korea [45] for high-throughput sequencing. GS FLX data processing was performed using Roche GS FLX software (v 2.9) in two stages, image processing and signal processing. Image processing involves normalization of raw images and generation of raw signals. In the signal processing stage, correction, filtering, and raw signal trimming were done prior to base calling with corresponding quality score of reads. Sequence reads from each sample were segregated with in-house script (Macrogen) using the tag (Barcode) sequences, and by matching the initial and final bases of the reads to the known tag sequences used in the preparation of the libraries.

### Bioinformatics analysis

Sequences were filtered for low quality bases and chimeric sequences. Only sequences of 100 bp., or more, were selected for final analysis. All sequence analyses were performed using QIIME version 1.8.0 [46] and open-reference operational taxonomic units (OTUs) picking strategy was employed. OTUs were picked based on 97% sequence similarity using UCLUST algorithm [47] and taxonomies were assigned against the well curated Silva_119 database [48]. The parameters used for OTU picking and taxonomic assignments are as follows: pick_otus.py -i all.merged.min100bp.fasta—-threads = 8 and assign_taxonomy.py -i rep_set.fna–r /Silva119_for_Qiime/rep_set/97/Silva _119_ rep _set 97.fna -t/Silva119_for_Qiime/taxonomy /97/ taxonomy_97_all_levels.txt -o taxonomy _results/ -e 0.01—uclust_similarity = 0.85. Sample specific OTUs were retrieved from all the OTUs and aligned against the same database by BLAST [49]. Finally, the taxonomic classification were plotted using metagenome analyser (MEGAN5) [50].

All 16S rRNA gene sequences were deposited in the European Nucleotide Archive (ENA- http://www.ebi.ac.uk/ena) under accession number PRJEB9174.

### Statistical analyses

A full model two factor permutational analysis of variance was run using Primer v. 6 with PERMANOVA add-on, to compare the bacterial communities between the hypobranchial gland and foot tissue of male and female *D*. *orbita*. Bray Curtis similarity matrices with a dummy value of 1 were generated from the untransformed OTU data at the genus level. Initial analyses were performed using the number of reads as a covariate to establish whether the unequal number of reads between samples influenced the outcomes. However, as the covariate was not significant (Pseudo F = 9.83, p = 0.96), the covariate was removed and the results are presented from the reduced two factor model. Additional analyses were also performed using a reduced data set excluding the low read samples (i.e. F1H, F2F and M2H) and these produced comparable results to the full data set (S1 Table). All PERMANOVA analyses were performed using 9999 permutations. Principal Coordinates Ordination (PCO) was undertaken to represent the data graphically. Similarity of Percentages (SIMPER) was run to establish which bacterial taxa contributed to the dissimilarity between the hypobranchial gland and foot tissue.

The DIVERSE function in PRIMER 6 was used to analyse the genus richness and diversity (Shannon’s H index), which was calculated from the relative abundance (% of reads) for each distinct OTU in each sample, but excluding the unassigned taxa. Univariate PERMANOVAs of genus richness and diversity were performed using Euclidean distance similarity matrices.

## Results

### Bacterial profiling of the hypobranchial gland and foot of *Dicathais orbita*

A total of 149,804 reads, with an average length of 436.301 base pairs, were obtained from the eight samples (four hypobranchial gland and four foot) of *D*. *orbita* (Table 1). Total acceptable reads, for operational taxonomic unit (OTU) assignment for the eight samples, ranged from 637 to 36,728 in the hypobranchial gland and foot (Table 1). At least one replicate from each sample type had > 15,000 reads. The total number of shared (non-overlapping) operational taxonomic units (OTUs) resulting from the bacterial profiling data set was 3585. The foot samples had a higher number of OTUs than the hypobranchial gland, across all taxonomic levels (Table 1).

**Table 1:** Summary of *Dicathais orbita* hypobranchial gland and foot tissue 16S rRNA bacterial profiling.

Tissue	Hypobranchial gland				Foot				
Gender	Female	Female	Male	Male	Female	Female	Male	Male	Total
Sample^1^	F1H	F2H	M1H	M2H	F2F	F3F	M2F	M3F	-
Reads	637	36,728	35,548	3,601	1,326	15,305	28,611	28,048	149,804
Total bases	253,914	15,930,139	15,597,012	1,544,017	597,192	6,681,905	12,563,818	12,191,707	65,359,704
Average read length	398.6	433.7	438.8	428.8	450.4	436.6	439.1	434.7	-
Operational taxonomic units^2^	50	526	1672	250	290	1055	1440	1496	3585
Number of Phyla	5	9	18	11	17	16	25	24	28
Number of Classes	11	21	36	21	30	32	55	53	65
Number of Orders	13	35	80	35	48	70	103	98	143
Number of Families	15	48	127	48	69	108	148	152	243
Number of Genera	17	66	221	71	124	204	277	288	443

^1^ The samples are labelled such that the first letter refers to the gender, the number to different replicate snails within each gender and the second letter to the tissue type.

^2^ OTUs are shared among multiple samples and are based on 97% sequence similarity criteria in the Silva_119 database.

**Fig 1:**
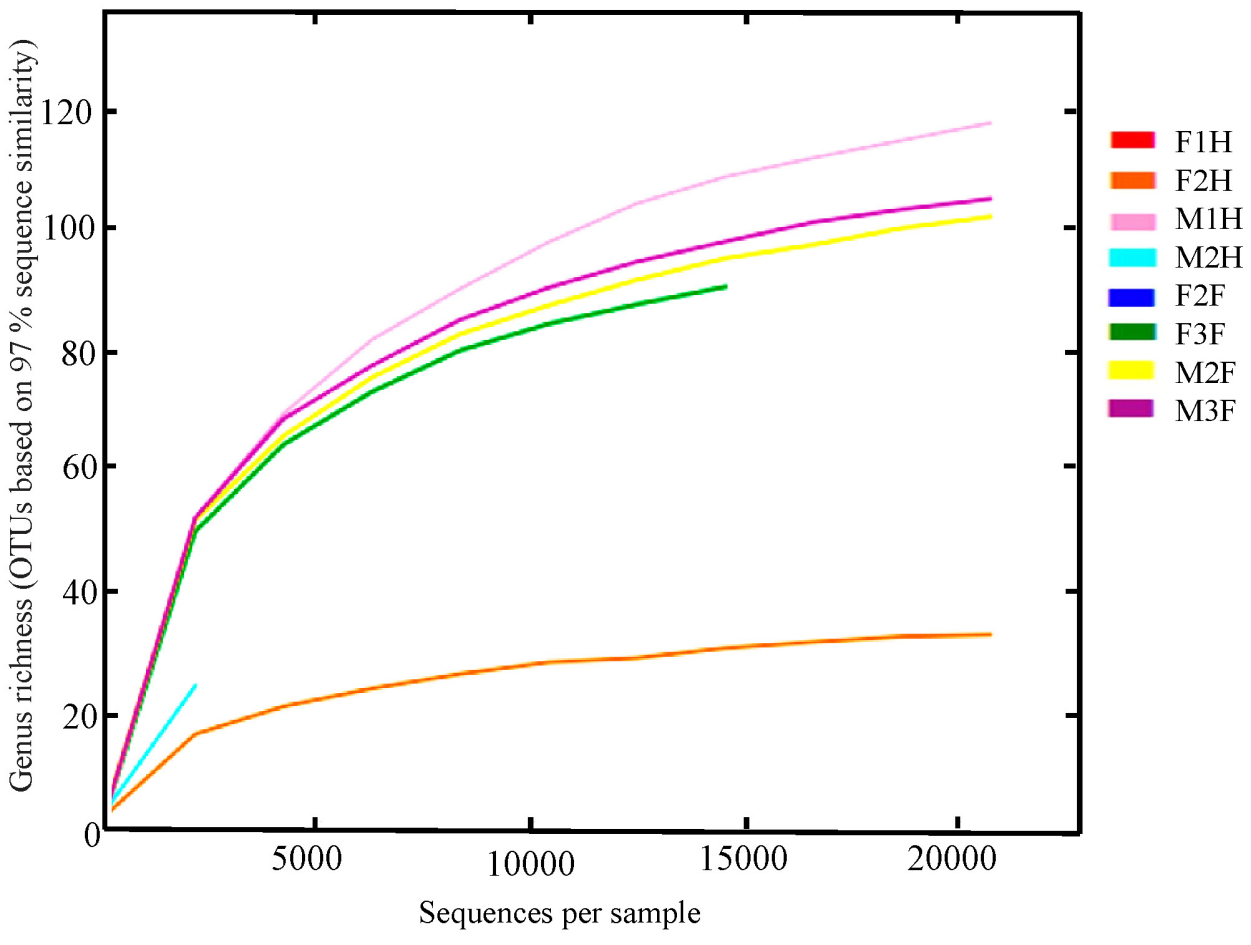
Alpha diversity showing the richness of bacterial community diversity within *Dicathais orbita* foot (F2F, F3F, M2F and M3F) and hypobranchial gland samples (F1H, F2H, M1H and M2H) (F = female; M = male). The phylogenetic diversity metric consists of genus richness based on 3585 observed OTUs at the 97% sequence similarity level and 443 possible observed genus. Sample with reads of more than 3000 are visible.

Rarefaction curves indicated the richness of bacterial taxa had not peaked at the maximum number of sequences read, with the exception of female hypobranchial gland 2, which reached an asymptote of < 70 bacterial genera after ~ 10,000 sequences (Table 1). The number of OTUs is likely to be highly under-represented in the other female hypobranchial gland sample (F1H = 17) and male hypobranchial gland 2 (M2H = 71, Table 1), due to the low number of sequence reads (Fig 1). The alpha diversity rarefaction plots also showed that the female hypobranchial gland had lower bacterial diversity than the male hypobranchial gland and foot samples of *D*. *orbita* (Fig 1).

### Bacterial taxonomic diversity of the hypobranchial gland and foot of *Dicathais orbita*

**Fig 2:**
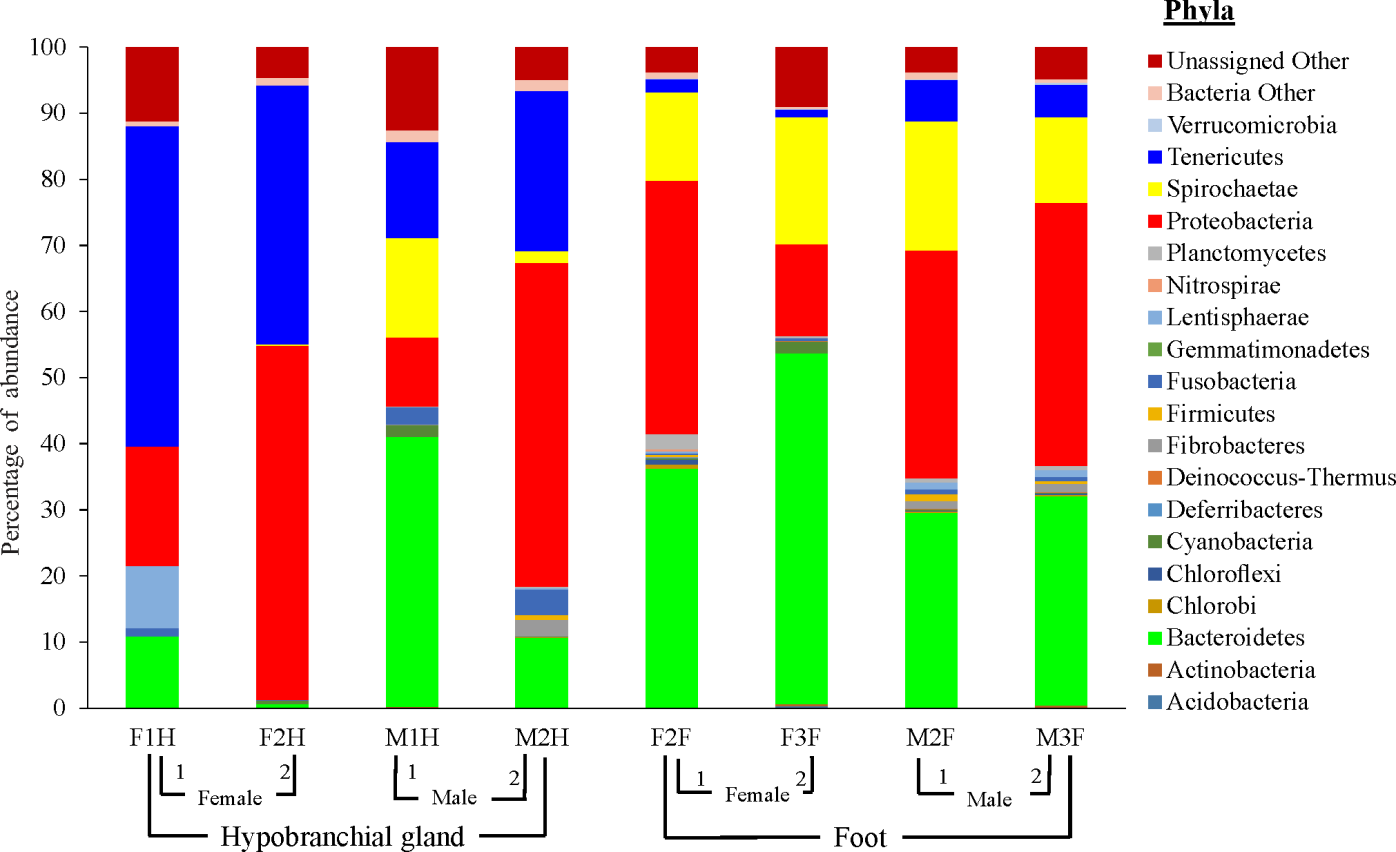
Phylum-level taxonomic diversity associated with the female (F) and male (M) hypobranchial gland (F1H, F2H, M1H and M2H) and foot (F2F, F3F, M2F and M3F) of *Dicathais orbita* bacterial profiling. All the minor phyla and unnamed, but previously identified bacterial phyla (such as BD1-5, CKC4, candidate division BRC1, OD1, OP8, SR1, TM7, SHA-109, and TM6) are grouped into “Bacteria Other”.

Altogether, 28 different bacterial phyla were observed in the bacterial profiling data set; however, only dominant phyla are presented (Fig 2). Bacterial groups that could not be assigned to any phyla equated to 6.8%. The dominant phylum was *Proteobacteria*, representing 32.2% of the bacterial abundance in all *D*. *orbita* samples (Fig 2). Bacteria from the phylum *Tenericutes* were more abundant in the hypobranchial gland compared to the foot (Fig 2). *Bacteriodetes* were more abundant in foot tissues than female hypobranchial glands (Fig 2). Bacteria from the phylum *Spirochaetes* were also more abundant in the foot than the hypobranchial gland samples (Fig 2).

**Fig 3:**
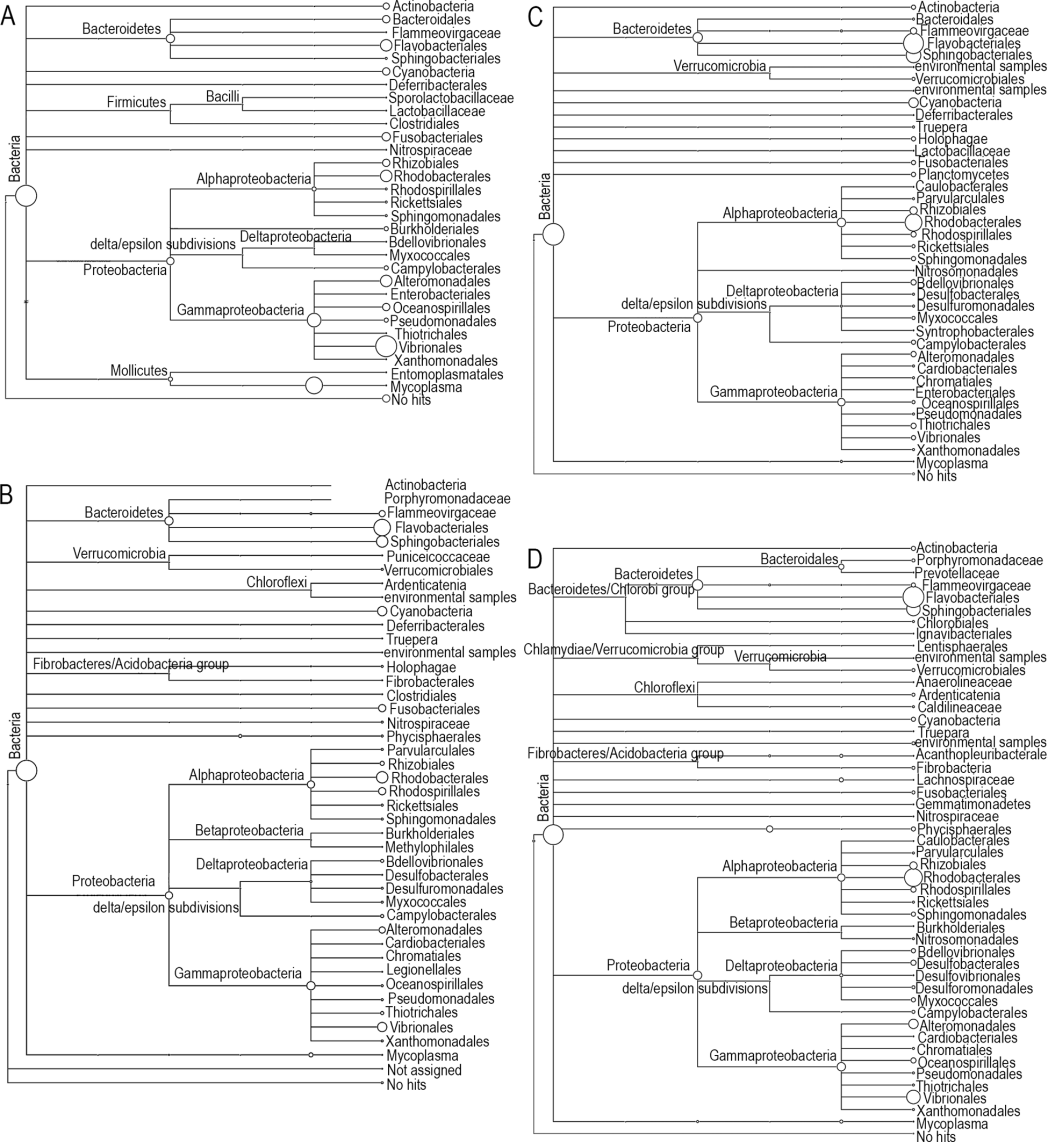
Phylogenetic tree of *Dicathais orbita* samples generated from 16S rRNA sequences by MEGAN. A = Female hypobranchial gland (F2H); B = Male hypobranchial gland (M1H); C = Female foot (F3F); D = Male foot (M3F). All these sample types have more than 15,000 reads.

Phylogenetic analysis revealed male foot tissue (M3F) had greater taxonomic diversity than the hypobranchial gland (Fig 3). *Flavobacteriales*, *Sphingobacteriales* and *Rhodobacterales* were more common in the foot, while *Vibrionales* was more dominant in hypobranchial gland (Fig 3). *Vibrionales* was the dominant order in the female hypobranchial gland (Fig 3A) and representatives from this order were observed in all samples of the foot and hypobranchial gland (Fig 3). *Mycoplasma*, in the phyla *Tenericutes*, was found to be more dominant in the hypobranchial gland when compared to *D*. *orbita* foot samples (Fig 3).

**Fig 4:**
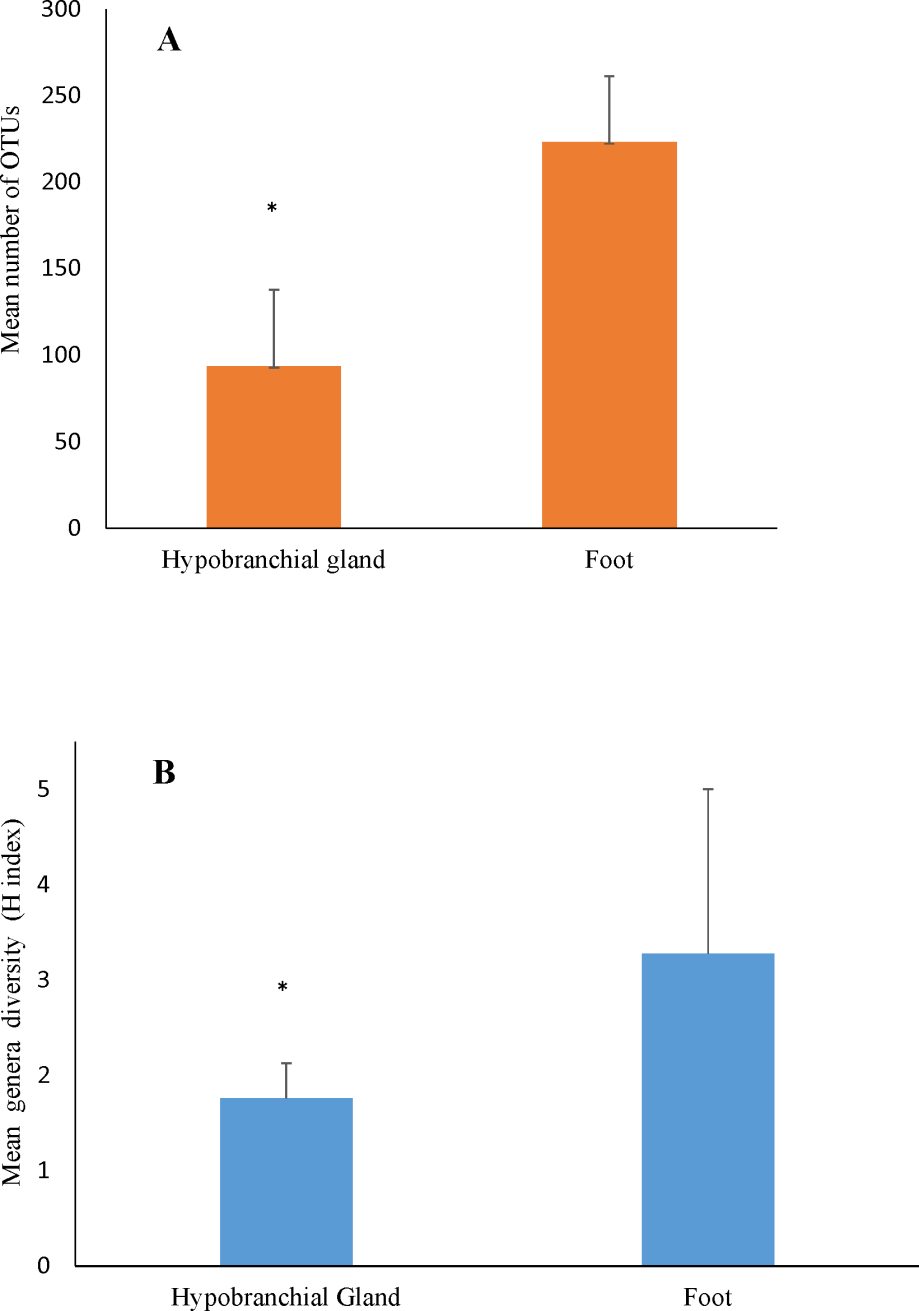
Mean (+s.e.) number of OTUs in the hypobranchial gland and foot tissue of *Dicathais orbita*, showing the mean proportion unique to individuals samples of foot and hypobranchial gland tissue. (A) = OTUs richness, (B) = H index/diversity.

Altogether, 443 known bacterial genera were identified in the foot and hypobranchial gland of the *D*. *orbita* bacterial profiling dataset, based on >97% sequence similarity. In total there were 169 distinct bacterial genera present in the foot,52 in the hypobranchial gland and 222 common bacterial genera between the foot and hypobranchial gland of *D*. *orbita*. On average, a higher number of distinct bacterial genera were recorded in the foot compared to hypobranchial gland samples (Fig 4A). Univariate PERMANOVA analysis confirmed there was significantly different genus richness between tissue types (Pseudo F = 8.54, p = 0.04). However, genus richness was not significantly different between genders (Pseudo F = 6.33, p = 0.06), and there was no interaction between gender and tissue type (Pseudo F = 2.49, p = 0.86).

Using Shannon’s diversity index to assess richness and evenness of the bacterial communities, higher diversity was consistently detected in the foot compared to the hypobranchial gland of *D*. *orbita* (Fig 4B). Univariate PERMANOVA analysis revealed genus diversity (Hˊ) was significantly higher in the foot than the hypobranchial gland (Pseudo F = 18.44, p = 0.01). There was no significant difference according to gender (Pseudo F = 3.71, p = 0.13), and no interaction between gender and tissue (Pseudo F = 1.79, p = 0.25).

### Bacterial community structure in the hypobranchial gland and foot of *Dicathais orbita*

**Fig 5:**
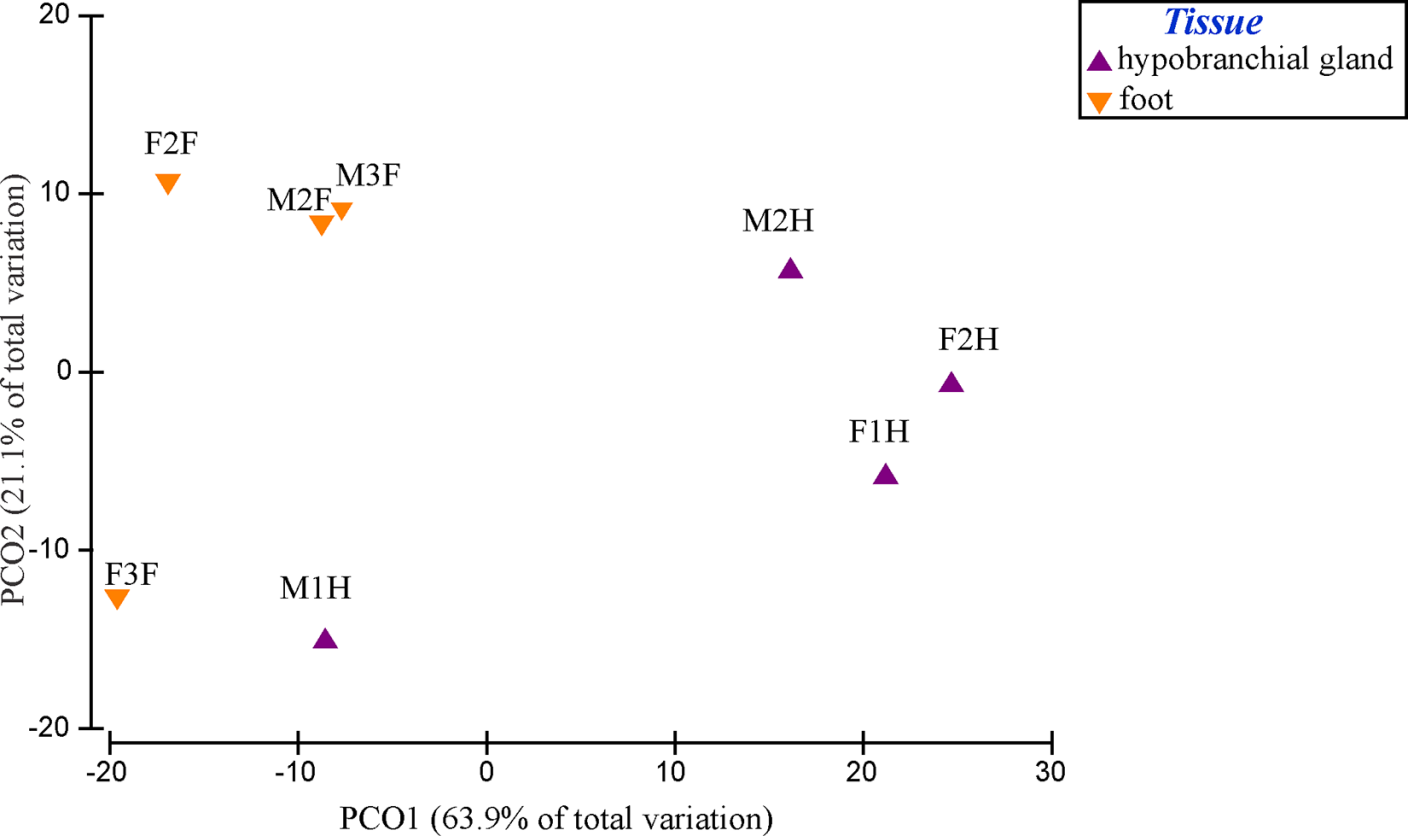
Principal Coordinates Ordination (PCO) of bacterial genus composition, based on a Bray Curtis similarity matrix of the relative abundance of OTUs at 97% sequence similarity level for the hypobranchial gland (purple) and foot (orange) of female (F) and male (M) *Dicathais orbita*.

Principal Coordinates Ordination (PCO) revealed separation of bacterial communities, based on genera level OTUs, between the hypobranchial gland and foot samples (Fig 5). The bacterial communities of hypobranchial gland samples were more variable and also showed separation between male and females, whereas foot samples clustered together on the left hand side of the plot (Fig 5). Multivariate analyses of genera OTUs associated with the hypobranchial gland and foot of *D*. *orbita*, revealed a significant difference between these tissues (Pseudo F = 5.46, p = 0.02). However, there was no significant difference according to gender (Pseudo F = 0.58, p = 0.67) and no interaction between gender and tissue (Pseudo F = 2.01, p = 0.17). Similar results were found when just the presence and absence of bacteria in the samples are considered (rather than relative abundance). Here, the PCO plot also revealed a general pattern of foot samples clustering separately and hypobranchial gland samples being more variable between the individual snails than foot samples (S1 Fig).

SIMPER (Similarity of percentages) analysis revealed high dissimilarity between the bacterial communities of the hypobranchial gland and foot of *D*. *orbita* (Table 2, Average dissimilarity = 68.51%). Of the 443 bacterial genera, four contributed approximately 50% of the dissimilarity between the tissues. *Vibrio* and *Mycoplasma* were more abundant in the hypobranchial gland, whereas *Chitinophagaceae* and *Spirochaeta* were more abundant in the foot (Table 2). A relatively small number of genera (e.g. *Mycoplasma*, *Vibrio*), along with an average of approximately 8% unassigned bacteria contributed to the similarity between hypobranchial gland samples of *D*. *orbita* (S2 Table). However, 30 diverse bacteria contributed to 90% of the similarity between the foot samples (S2 Table).

**Table 2:** Similarity of percentages (SIMPER) analysis showing the bacterial genus that contribute most to the differences between hypobranchial gland and foot of *Dicathais orbita* (Average dissimilarity = 68.51).

Genus	Group Hypobranchial gland Av. Abundance	Group Foot Av. Abundance	Av. Diss.	Diss./ SD	Contrib. %	Cum. %
*Mycoplasma*	0.32	0.04	13.99	2.03	20.42	20.42
*Vibrio*	0.27	0.09	10.83	1.27	15.81	36.24
*Chitinophagaceae*; Other	0.08	0.15	8.07	1.29	11.78	48.02
*Spirochaeta*	0.04	0.16	6.20	1.96	9.05	57.07
*Owenweeksia*	0.01	0.07	3.29	2.16	4.80	61.87
Unassigned; Other	0.08	0.05	2.03	1.26	2.97	64.84
*Rhodobacteraceae*; Other	0.01	0.05	1.81	1.98	2.65	67.49
*Oligosphaeria*; uncultured bacterium	0.02	0.01	1.29	0.68	1.89	69.38
*Marinilabiaceae*; uncultured	0.02	0.01	1.08	1.32	1.57	70.95
*Rhodobacteraceae*; uncultured	0.01	0.03	1.05	1.94	1.54	72.48
*Flavobacteriaceae*; uncultured	0.01	0.03	0.98	1.55	1.43	73.92
*Propionigenium*	0.02	0	0.72	1.49	1.06	74.97
*Roseovarius*	0	0.01	0.72	1.70	1.05	76.02
*Flavobacteriaceae*; Other	0.01	0.02	0.64	1.66	0.93	76.95
*Marinifilum*	0.01	0	0.59	1.03	0.87	77.82
*Saprospiraceae*; uncultured	0	0.01	0.52	2.46	0.77	78.59
Bacteria; Other	0.01	0	0.50	2.50	0.72	79.31
*Bacteroidetes*; Other	0.01	0	0.49	0.75	0.71	80.02
*Fibrobacteria Incertae Sedis*; possible genus03	0.01	0.01	0.47	1.13	0.69	80.71
*Flammeovirgaceae*; Other	0	0.01	0.37	1.06	0.54	81.25
*Aureispira*	0	0.01	0.35	1.69	0.51	81.75

### Biosynthetic capabilities of the bacterial symbionts

**Fig 6:**
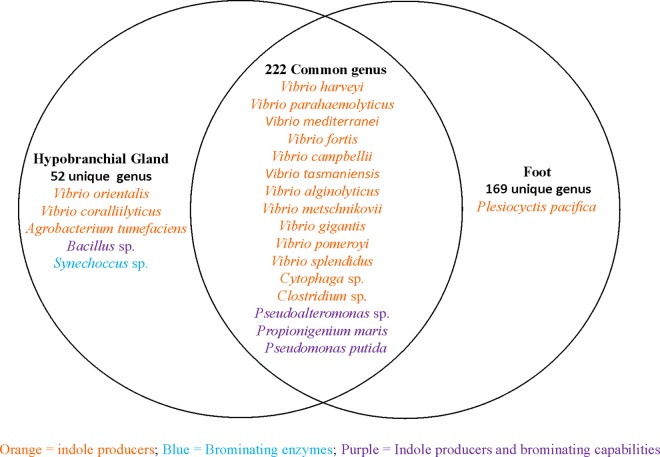
Venn diagram showing shared and non-shared bacterial species between the hypobranchial gland and foot of *Dicathais orbita*. The number of species that have biosynthetic capabilities relevant to Tyrian purple production are highlighted in different colours (Orange = indole producers; Blue = brominating enzymes; Purple = indole producers and brominating capabilities).

Of the possible 443 bacterial genera identified from the tissues of *D*. *orbita*, only 22 bacterial species are known to have biosynthetic capabilities directly relevant to Tyrian purple precursor biosynthesis (Fig 6, S3 Table). A greater proportion of the bacteria found only in the hypobranchial glands (9.6%) were found to have indole and/ or brominating capabilities compared to those only found in the foot (0.6%, Fig 6). There were 21 indole producing species detected across 9 genera (Fig 6) and the majority of these were *Vibrio* spp. common to both the foot and hypobranchial gland samples (Fig 6). Three species were detected that are known to produce both indoles and brominated secondary metabolites and a further three species produce bromoperoxidase enzymes (Fig 6, S3 Table). More specifically, bacteria from three genera that were detected more frequently in the hypobranchial gland, namely *Bacillus*, *Pseudomonas* and *Synechococcus*, are known to produce bromoperoxidase (S3 Table). *Pseudomonas* spp., and several other bacteria found in the hypobranchial gland, are also known to produce oxidised sulphur metabolites, whereas three sulphur reducing bacteria were found exclusively in the foot tissue (S3 Table).

## Discussion

This study determined differences in bacterial community composition in the Tyrian purple producing hypobranchial gland and the non-biosynthetic foot tissues of the muricid mollusc, *D*. *orbita*. Bacterial taxa representing 3585 OTUs from 28 different phyla, 243 families and 443 genera (Table 1) were observed in the bacterial profiling data set of *D*. *orbita*. Phylogenetic analysis highlighted the presence of more complex bacterial communities in the foot compared to the hypobranchial gland, and this was supported by significantly lower OTU richness and diversity in the gland than the foot. PCO and multivariate analysis revealed significantly different bacterial community structure between the two tissues, and dissimilarity analysis revealed a higher abundance of *Vibrio*, *Mycoplasma* and unassigned bacteria in the biosynthetic hypobranchial gland. Consistent with previous culture studies [39], *Vibrios* were the dominant indole producing bacteria detected. However, 16S rDNA bacterial profiling also revealed the presence of bromoperoxidase producing bacteria such as *Bacillus*, *Pseudomonas* and *Synechococcus*, and bacteria known to produce brominated secondary metabolites, such as *Pseudoalteromonas* and *Propionigenium*, in the Tyrian purple producing gland.

The taxonomic diversity of symbiotic bacteria, with a dominance of *Proteobacteria* in *D*. *orbita*, is comparable with previous 16S rDNA analyses of marine mollusc associated bacteria. Metagenomic (16S rDNA) bacterial diversity studies of the molluscan sea slug, *Elysia chlorotica*, from a total number of reads among samples ranging from 4601 to 11374, produced 199 to 889 OTUs derived from 5 to 9 distinct phyla [51]. Another metagenomic study of the 16S rRNA gene of a bivalve mollusc (internal body parts without the shell) resulted in the discovery of 3553 OTUs from 44 phyla, in which *Proteobacteria* was found to be the most abundant phylum [52]. The metagenome of the digestive tract of a marine limpet also revealed diverse microbial communities with the most dominant phylum being *Proteobacteria* [53]. Indeed, *Proteobacteria* accounts for more than 40% of all known prokaryotic genera [54] and is the dominant bacterial phyla reported from other marine invertebrate taxa, including the sponge *Halichondria* sp. [55], the coral *Porites astreoides* [56] and the oyster *Crassostrea* sp.[57].

Significantly different bacterial community structure, with a higher richness and diversity of OTUs, was observed in the foot relative to the hypobranchial gland samples of *D*. *orbita* (Figs 3–5). This is consistent with a previous study on heterotrophic culturable bacteria, where no bacterial biochemical activity was detected in the homogenised glands and significantly fewer species were isolated from swabs of the hypobranchial gland in comparison to the foot tissue of *D*. *orbita* [39]. The lower Shannon’s diversity index from the hypobranchial gland coupled with the SIMPER analyses imply the bacterial community in this gland is dominated by two abundant symbiotic genera (i.e. *Mycoplasma*, *Vibrio*), some unassigned, possibly novel bacteria and a larger number of genera detected in low abundance, many of which may be rare opportunists or contaminants. This pattern of bacterial diversity is consistent with a highly specialised internal environment; indeed the hypobranchial gland has a low pH [39] and produces secretions containing antibacterial sulphated mucopolysaccharides and brominated indoles that would be expected to kill the majority of opportunistic bacteria [41, 58]. *Mycoplasma*, the best known genus in the class *Mollicutes*, are common parasites in marine organisms and can persist in extreme environments including low pH and low oxygen [59, 60]. They lack a cell wall and are unaffected by many antibiotics [61]. They are also common laboratory contaminants [62], but may exist as parasites or commensals within the hypobranchial gland, which provides a rich source of carbohydrates. To establish the potential for vertical or horizontal transmission of the bacterial symbionts in *D*. *orbita*, future bacterial profiling studies could include samples of the egg capsules, water and benthic substrate for comparison. Interestingly, a preliminary study on the culturable heterotrophic bacteria from *D*. *orbita* tissues identified at least one indole producing bacteria common to the hypobranchial gland and egg capsules [39]. The precursors of Tyrian purple are found in the egg capsules [3], as well as the female capsule gland, which lies adjacent to the hypobranchical gland in *D*. *orbita* [1, 63] suggesting a potential role of vertical transmission of biosynthetic symbiotic bacteria.

The dominance of *Vibrionaceae* in the hypobranchial glands of *D*. *orbita* implies these bacteria are selectively retained or are able to multiply within this unusual mucus producing organ. *Vibrios* are commonly associated with marine organisms [64–66] and species such as *V*. *parahaemolyticus*, *V*. *orientalis* and *V*. *campbellii* are pathogens of marine invertebrates [67–69] with *V*. *orientalis*, *V*. *harveyi*, *V*. *coralliilyticus* and *V*. *splendidus* being specific mollusc pathogens [68, 70–72]. However, *Vibrio* species can also be endosymbionts such as those found in the viscera of the muricid, *Nucella lapillus*, and mucus of the sea slug, *Elysia rufescens* [73, 74], as well as *V*. *fischeri*, which is found in squid bioluminescent organs [19]. *Vibrio* species are known, not only for their symbiotic relationships with marine molluscs, but also for the production of important secondary metabolites [75–77]. A previous study successfully cultured three indole producing *Vibrio* species from the biosynthetic organs of *D*. *orbita* [39]. Many additional indole producing *Vibrio* species from the hypobranchial gland of *D*. *orbita* were identified in this study (S3 Table). The relatively high concentration of these *Vibrios* in the hypobranchial gland, and their capacity for indole synthesis, suggest they may contribute to Tyrian purple precursor synthesis in the hypobranchial glands of Muricidae. Indole producing *Vibrio* species, including *V*. *orientalis* and *V*. *coralliilyticus* were found exclusively in the hypobranchial gland, but not in the foot. A range of other bacterial genera that produce indoles were also detected in the hypobranchial gland. These include *Bacillus*, [78] *Propionigenium*, [79] and *Pseudomonas*, which produce indoles such as indole-3-acetic acid [80]. Hence, indole precursors may be opportunistically acquired from more than one bacterial species for Tyrian purple production in muricids.

It has been suggested bromoperoxidase plays a role in Tyrian purple biosynthesis through the addition of bromine to the 6-position of tyrindoxyl sulphate [81, 82]. This is supported by evidence of bromoperoxidase activity in *Trunculariopsis (Murex) trunculus* hypobranchial gland homogenates [17] and histochemical sections from *D*. *orbita* hypobranchial and rectal glands [18, 83]. Several bacterial genera were detected in our bacterial profiling studies that are known to produce bromoperoxidase (S3 Table), including *Pseudomonas* [24] and bacteria of the *Bacillaceae* family [84]. The cyanobacterium, *Synechococcus* produce vanadium dependent bromoperoxidase [31]; this enzyme is implicated in the biosynthesis of marine halogenated natural products of pharmacological importance [85] and can also react with indole to produce region specific brominated indole products [85, 86]. *Bacillus*, *Pseudomonas* and *Synechococcus* were all present in the hypobranchial gland of *D*. *orbita* and these bromoperoxidase producing bacterial genera are the priorities for future targeted culture work to further investigate their role in Tyrian purple production in *D*. *orbita*. Future studies could also apply functional metagenomics approaches to uncover the brominating enzymology associated with the hypobranchial glands of Muricidae by screening specifically for bromoperoxidase and brominase genes [87].

Several other marine bacteria found in the hypobranchial glands are known to produce halogenases and could provide an alternative path for brominating indole precursors of Tyrian purple. Halogenating enzymes such as brominases, responsible for the synthesis of polybrominated metabolites, including phenol and imidazole structures, have been identified in marine bacteria [87]. Tribromoimidazole, a brominated secondary metabolite found within the eggs of muricid molluscs, may be produced by brominase activity [88]. *Pseudomonas* sp. also produce halogenase enzymes [21] and we detected *Pseudomonas* spp. in the hypobranchical glands of *D*. *orbita*. Other marine studies have isolated a tryptophan 6-halogenase with brominating activity from *Streptomyces* sp. [23] and a novel halogenase gene from *Psychrobacter* sp. (associated with the marine sponge *Crambe crambe*) [22], a genus that was also detected in our *D*. *orbita* study. Other bacteria detected in the hypobranchial gland of *D*. *orbita*, including *Vibrio*, and *Pseudoalteromonas*, have previously been found to produce brominated secondary metabolites (S3 Table). For example, *Vibrio* sp. (strain KMM-81-1) associated with the marine sponge (*Dysidea* sp.) produces brominated secondary metabolites [89], and several species of *Pseudomonas* produce brominated nitrophenyl pyrrole compounds [90, 91], while *Pseudoalteromonas* sp. can produce pentabromopseudilin and bromophene [92]. However, these bacteria were not specifically associated with the hypobranchial glands of *D*. *orbita* and thus appear less likely candidates for providing tissue localised precursors to Tyrian purple.

It is possible bacteria may be responsible for several steps which occur early in the Tyrian purple biosynthetic pathway. Enzymes such as sulphur transferase and sulphur reductase may be involved in contributing the methane thiol group on the indole ring of tyrindoxyl sulfate. High concentrations of mercaptan and dimethyl disulfide are present in muricid hypobranchial glands that produce Tyrian purple [1, 4, 93]. *Pseudomonas* found in the hypobranchial gland is known to utilize dimethyl disulfide [94]. Several bacteria that metabolise sulphur, such as *V*. *orientalis* and *V*. *coralliilyticus* are exclusively found in the hypobranchial gland and also utilize dimethylsulfoniopropionate, an organosulfur compound that produces dimethyl sulphide and methanethiol as a breakdown product [95, 96]. *Sulfitobacter mediterraneus* was detected in the foot and hypobranchial gland and is a sulfite-oxidizing bacteria [97] that may catalyse the production of indoxyl sulfate. Thus, it is possible the various sulphur metabolizing bacteria found in the hypobranchial gland play important roles in Tyrian purple precursor production.

A difference in the 16s rRNA bacterial profiles of male and female hypobranchial glands was expected on the basis that previous chemical studies have suggested a difference in the oxidation and reduction state of indole dye precursors in male and female hypobranchial glands. Specifically, the female glands were found to contain higher amounts of reduced methanethiol derivatised indoles, such as tyrindoleninone and tyriverdin, whereas males contained more oxidised end-products 6-bromoisatin and 6,6’dibromoindirubin [1]. This could imply the presence of sulfur-reducing bacteria in the female hypobranchial glands, although we actually found more evidence for known sulfur-reducing bacteria in the foot tissue (S3 Table) and none were unique to the female hypobranchial gland. Nevertheless, the reducing environment of the female gland could explain why the bacterial community structure was noticeably more distinct from the foot communities than the male glands (Fig 5) and the tendency towards lower phylogenetic complexity in the female compared to male hypobranchial glands (Table 1, Fig 3). However, consistent with a previous culture based study [39], there were no significant difference in the bacterial communities isolated from male and female samples and no interaction between tissue and gender. In both studies the lack of a significant gender effect could be influenced by a consistent bacterial community structure within the foot tissues and low power to detect a gender difference, specifically in the hypobranchial glands, due to relatively high variability and low replication of male and female samples within this tissue type (e.g. Fig 5 PCO). Consequently, future functional metagenomics studies aimed specifically towards examining the sulphur metabolising bacteria in male and female hypobranchicial glands of Muricidae are warranted.

Overall, a larger number of bacterial taxa were found in the foot compared with the hypobranchial gland of *D*. *orbita*, however, a higher abundance of *Vibrio* and some unique microbial symbionts were observed in the hypobranchial gland. Some of the bacteria identified in the hypobranchial gland are known to produce indole and bromoperoxidase or other enzymes which may contribute to Tyrian purple precursor synthesis. Future studies will aim to culture these microbial symbionts associated with the hypobranchial gland and further analysis will be undertaken to identify genes that may be associated with Tyrian purple precursor production.

## Supporting Information

S1 Fig Principal Coordinates Ordination (PCO) of bacterial genus associated with hypobranchial gland (purple) and foot (orange) of female (F) and male (M) *Dicathais orbita* after presence/ absence transformation. (TIF)

S1 Table Summary of statistical analyses for genus level using a reduced data set (F2H, M1H, F3F, M2F and M3F) excluding samples with low number of reads (F1H, M2H and F2F). Univariate PERMANOVA was performed on Euclidean distance similarity matrices for genus level OTU richness and diversity, whereas multivariate PERMANOVA was performed using Bray-Curtis similarity matrices for community composition based on the number of reads.
(DOCX)

S2 Table Similarity of percentages (SIMPER) analysis showing the bacterial genus that contribute the most to the similarity in A) hypobranchial gland (Average similarity: 45.47) and B) the foot of *Dicathais orbita* (Average similarity: 60.14). (DOCX)

S3 Table *Dicathais orbita* associated bacteria that have been previously shown to produce indoles, brominated secondary metabolites or enzymes associated with their biosynthesis or sulphur metabolizing bacteria. (DOCX)
