# Supplementary figures and images for: Characterization of Bacterial Communities Associated with the Tyrian Purple Producing Gland in a Marine Gastropod
Source: PLoS One. 2015 Oct 21;10(10):e0140725. doi: 10.1371/journal.pone.0140725 (PMC4619447; doi:10.1371/journal.pone.0140725)

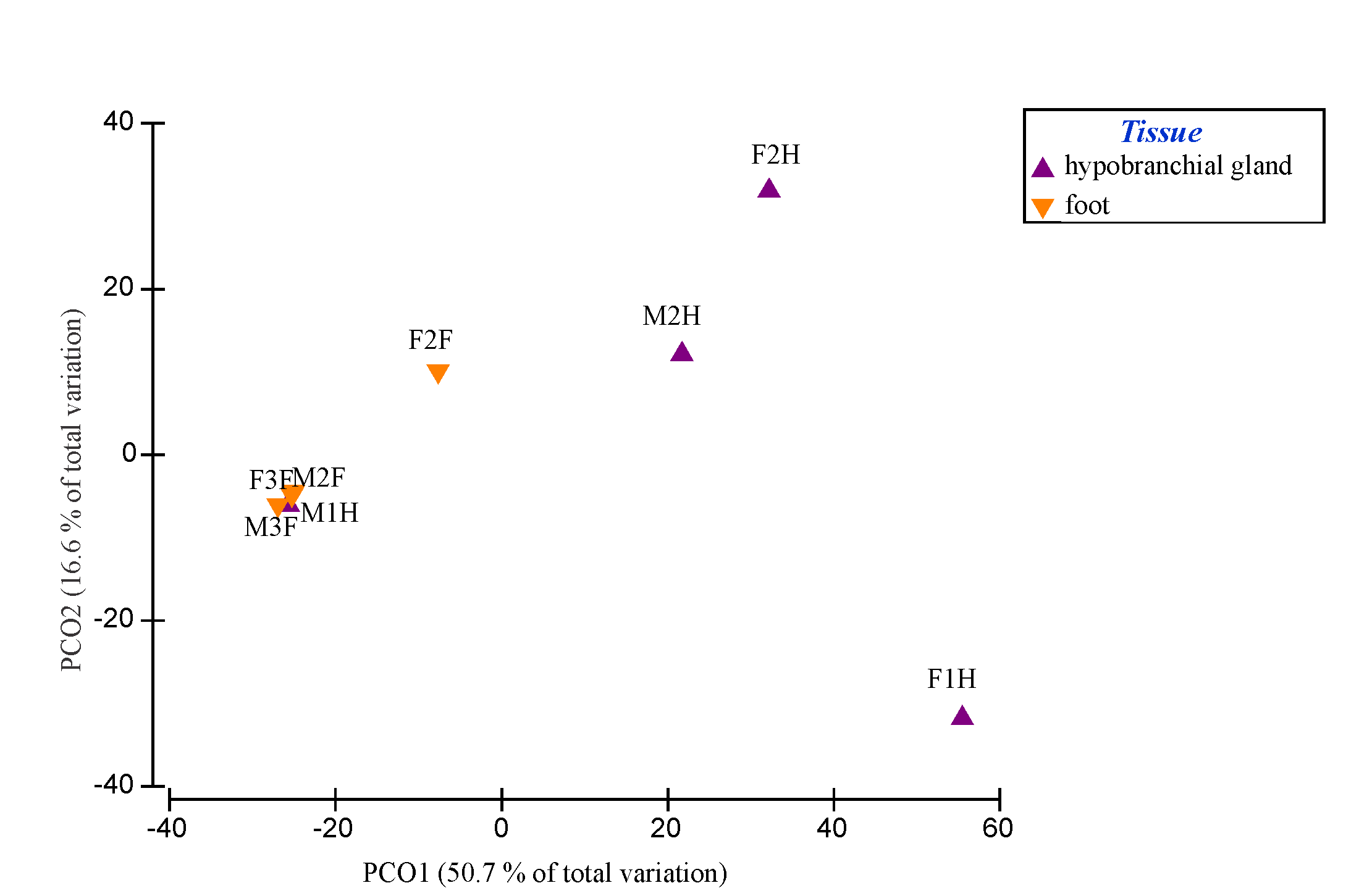

Supplement: S1 Fig — (TIF) [file pone.0140725.s001.tif]
