# Supplementary material for: Characterization of Bacterial Communities Associated with the Tyrian Purple Producing Gland in a Marine Gastropod
Source: PLoS One. 2015 Oct 21;10(10):e0140725. doi: 10.1371/journal.pone.0140725 (PMC4619447; doi:10.1371/journal.pone.0140725)
Supplement: S1 Table — Univariate PERMANOVA was performed on Euclidean distance similarity matrices for genus level OTU richness and diversity, whereas multivariate PERMANOVA was performed using Bray-Curtis similarity matrices for community composition based on the number of reads. (DOCX) [file pone.0140725.s002.docx]

**S1 Table: Summary of statistical analyses for genus level using a reduced data set (F2H, M1H, F3F, M2F and M3F) excluding samples with low number of reads (F1H, M2H and F2F). Univariate PERMANOVA was performed on Euclidean distance similarity matrices for species richness and diversity, whereas multivariate PERMANOVA was performed using Bray-Curtis similarity matrices for community composition based on the number of reads.**

| **Analysis** | **Tissues** | | **Gender** | | **Tissue and Gender interaction** | |
| --- | --- | --- | --- | --- | --- | --- |
|  | Pseudo F value | P (perm) value | Pseudo F value | P (perm) value | Pseudo F value | P (perm) value |
| Community composition | 29.99 | 0.03 | 11.66 | 0.09 | 21.58 | 0.07 |
| H diversity | 1907.40 | 0.03 | 226.63 | 0.10 | 582.74 | 0.07 |
| Richness | 66.31 | 0.06 | 467.11 | 0.03 | 9.56 | 0.84 |
