# Supplementary material for: Characterization of Bacterial Communities Associated with the Tyrian Purple Producing Gland in a Marine Gastropod
Source: PLoS One. 2015 Oct 21;10(10):e0140725. doi: 10.1371/journal.pone.0140725 (PMC4619447; doi:10.1371/journal.pone.0140725)
Supplement: S2 Table — (DOCX) [file pone.0140725.s003.docx]

**S2A Table: Percentage of similarity (SIMPER) analysis showing genus that contribute to similarity in replicate *D. orbita* hypobranchial gland samples (Average similarity: 45.47).**

| **Genus** | **Av. Abund** | **Av. Sim** | **Sim/ SD** | **Contrib. %** | **Cum. %** |
| --- | --- | --- | --- | --- | --- |
| *Mycoplasma* | 0.32 | 21.81 | 2.24 | 47.97 | 47.97 |
| *Vibrio* | 0.27 | 13.09 | 0.99 | 28.79 | 76.76 |
| Unassigned;Other;Other;Other;Other;Other | 0.08 | 5.81 | 2.19 | 12.77 | 89.54 |
| Bacteria;Other;Other;Other;Other;Other | 0.01 | 0.93 | 4.18 | 2.04 | 91.57 |

**S2B Table: Percentage of similarity (SIMPER) analysis showing genus that contribute to similarity in replicate *D. orbita* foot samples (Average similarity: 60.14).**

| **Genus** | **Av. Abund** | **Av. Sim** | **Sim/ SD** | **Contrib. %** | **Cum. %** |
| --- | --- | --- | --- | --- | --- |
| *Spirochaeta* | 0.16 | 13.99 | 5.75 | 23.26 | 23.26 |
| *Chitinophagaceae;*Other | 0.15 | 7.2 | 6.53 | 11.97 | 35.23 |
| *Owenweeksia* | 0.07 | 5.51 | 1.59 | 9.16 | 44.39 |
| Unassigned;Other;Other;Other;Other;Other | 0.05 | 3.96 | 8.86 | 6.58 | 50.97 |
| *Vibrio* | 0.09 | 3.84 | 0.74 | 6.39 | 57.36 |
| *Rhodobacteraceae*;Other | 0.05 | 3.72 | 3.04 | 6.18 | 63.54 |
| *Rhodobacteraceae*;uncultured | 0.03 | 2.82 | 18.13 | 4.69 | 68.24 |
| *Mycoplasma* | 0.04 | 2.05 | 1.44 | 3.41 | 71.65 |
| *Flavobacteriaceae*;uncultured | 0.03 | 1.73 | 2.68 | 2.88 | 74.53 |
| *Flavobacteriaceae*;Other | 0.02 | 1.71 | 17.95 | 2.84 | 77.36 |
| *Saprospiraceae*;uncultured | 0.01 | 0.97 | 8.18 | 1.61 | 78.98 |
| *Roseovarius* | 0.01 | 0.93 | 0.96 | 1.54 | 80.52 |
| *Roseobacter* clade NAC11-7 lineage | 0.01 | 0.61 | 6.53 | 1.02 | 81.54 |
| *Polaribacter* | 0.01 | 0.45 | 2.72 | 0.75 | 82.29 |
| *Aureispira* | 0.01 | 0.42 | 1.22 | 0.69 | 82.98 |
| *Colwellia* | 0.01 | 0.4 | 1.15 | 0.67 | 83.65 |
| *Marinilabiaceae*; uncultured | 0.01 | 0.4 | 0.78 | 0.66 | 84.31 |
| *Aquimarina* | 0.01 | 0.38 | 4.2 | 0.63 | 84.94 |
| *Lutibacter* | 0.01 | 0.35 | 1.16 | 0.58 | 85.52 |
| *Propionigenium* | 0 | 0.32 | 3.03 | 0.52 | 86.04 |
| *Tenacibaculum* | 0 | 0.31 | 3.16 | 0.52 | 86.57 |
| *Cohaesibacter* | 0.01 | 0.3 | 0.93 | 0.5 | 87.07 |
| *Arcobacter* | 0 | 0.29 | 2.42 | 0.48 | 87.54 |
| *Maribacter* | 0 | 0.28 | 0.91 | 0.47 | 88.01 |
| *Psychroserpens* | 0 | 0.26 | 1.15 | 0.44 | 88.45 |
| *Sulfitobacter* | 0 | 0.26 | 3.05 | 0.43 | 88.88 |
| *Fibrobacteria* Incertae Sedis; possible genus 03 | 0.01 | 0.22 | 0.47 | 0.37 | 89.26 |
| *Portibacter* | 0 | 0.22 | 9.03 | 0.37 | 89.62 |
| *Flavobacteriales*;Other | 0 | 0.21 | 2.89 | 0.36 | 89.98 |
| *Oligosphaeria;*uncultured bacterium | 0.01 | 0.21 | 0.57 | 0.35 | 90.33 |
