# Supplementary material for: Characterization of Bacterial Communities Associated with the Tyrian Purple Producing Gland in a Marine Gastropod
Source: PLoS One. 2015 Oct 21;10(10):e0140725. doi: 10.1371/journal.pone.0140725 (PMC4619447; doi:10.1371/journal.pone.0140725)
Supplement: S3 Table — (DOCX) [file pone.0140725.s004.docx]

**S3 Table: *Dicathais orbita* associated bacteria that have been previously shown to produce indoles, brominated secondary metabolites or enzymes associated with their biosynthesis or sulphur metabolizing bacteria**

| **Bacteria** | **Foot** | **Hypobranchial gland** | **Indole** | **Bromoperoxidase** | **Brominated compounds** | **Sulphur metabolism** |
| --- | --- | --- | --- | --- | --- | --- |
| *Vibrio parahaemolyticus* | 3/4 | 3/4 | Yes [[1](#_ENREF_1)] | - | - | - |
| *Vibrio orientalis* | 0/4 | 1/4 | Yes [[2](#_ENREF_2)] | - | - | Yes, cleave dimethylsulfoniopropionate (DMSP) [[3](#_ENREF_3)] |
| *Vibrio mediterranei* | 4/4 | 3/4 | Yes [[4](#_ENREF_4)] | - | - | - |
| *Vibrio fortis* | 2/4 | 2/4 | Yes [[5](#_ENREF_5)] | - | - | - |
| *Vibrio campbellii* | 3/4 | 1/4 | Yes [[6](#_ENREF_6)] | - | - | - |
| *Vibrio coralliilyticus* | 0/4 | 2/4 | Yes [[7](#_ENREF_7)] | - | - | Yes, used dimethylsulfoniopropionate [[8](#_ENREF_8)] |
| *Vibrio tasmaniensis* | 2/4 | 4/4 | Yes [[9](#_ENREF_9)] | - | - | - |
| *Vibrio alginolyticus* | 3/4 | 2/4 | Yes[[9](#_ENREF_9)] | - | - | - |
| *Vibrio metschnikovii* | 3/4 | 2/4 | Yes [[10](#_ENREF_10)] |  |  |  |
| *Vibrio gigantis* | 1/4 | 3/4 | Yes [[11](#_ENREF_11)] | - | - | - |
| *Vibrio harveyi* | 3/4 | 2/4 | Yes [[12](#_ENREF_12)] | - | - | - |
| *Vibrio pomeroyi* | 1/4 | 1/4 | Yes [[13](#_ENREF_13)] | - | - | - |
| *Vibrio splendidus* | 1/4 | 2/4 | Yes [[2](#_ENREF_2)] | - | - | - |
| *Desulfotalea arctica* | 2/4 | 1/4 | - | - | - | Yes, sulphur reducing bacteria [[14](#_ENREF_14)] |
| *Cytophaga* sp. | 3/4 | 3/4 | Yes [[15](#_ENREF_15)] | - | - | - |
| *Propionigenium maris* | 4/4 | 4/4 | Yes [[16](#_ENREF_16)] |  | Yes, 2,4,6-tribromophenol (TBP) to monobromophenols [[17](#_ENREF_17)] | Yes, succinate decarboxylation [[16](#_ENREF_16)] |
| *Clostridium* sp. | 1/4 | 1/4 | Yes [[18](#_ENREF_18)] | - | - | Yes, produces elemental sulphur from thiosulphate[[19](#_ENREF_19)] |
| *Bacillus* sp. | 0/4 | 1/4 | Yes, indole 3 acetic [[20](#_ENREF_20)] | Yes [[21](#_ENREF_21)] | - | - |
| *Desulfobulbus mediterraneus* | 1/4 | 0/4 | - | - | - | Yes, sulphur reducing bacteria [[22](#_ENREF_22)] |
| *Desulfoluna spongiiphila* | 1/4 | 0/4 | - | - | - | Yes, dehalogenating bacterium [[23](#_ENREF_23)] |
| *Desulfoluna butyratoxydans* | 1/4 | 0/4 | - | - | - | Yes, sulphur reducing bacteria [[24](#_ENREF_24)] |
| *Rhodovulum sulfidophilum* | 3/4 | 0/4 | - | - | - | Yes, oxidation of thiosulfate and sulphide [[25](#_ENREF_25)] |
| *Sulfitobacter mediterraneus* | 4/4 | 3/4 | - | - | - | Yes, sulfite-oxidizing [[26](#_ENREF_26)] |
| *Ruegeria pomeroyi* | 1/4 | 1/4 | - | - | - | Yes, dimethylsulphoniopropionate (DMSP)[[27](#_ENREF_27)] |
| *Synechococcus* sp*.* | 0/4 | 1/4 |  | Yes [[28](#_ENREF_28)] | - | - |
| *Desulfobulbus mediterraneus* | 1/4 | 0/4 | - | - | - | Yes, sulphur reducing bacteria [[22](#_ENREF_22)] |
| *Agrobacterium tumefaciens* | 0/4 | 1/4 | Yes, indole acetic acid [[29](#_ENREF_29)] | - | - | - |
| *Plesiocystis pacifica* | 3/4 | 0/4 | Yes [[30](#_ENREF_30)] | - | - | - |
| *Pseudomonas putida* | 1/4 | 2/4 | Yes, indole-3-acetic acid [[31](#_ENREF_31)] | Yes [[32](#_ENREF_32)] | Yes, dibromoethenes [[33](#_ENREF_33)] | Yes, oxidized to methanesulfonate [[34](#_ENREF_34)] |
| *Pseudoalteromonas* sp. | 4/4 | 2/4 | Yes [[35](#_ENREF_35)] | - | Yes, 2,4-dibromo-6-chlorophenol [[36](#_ENREF_36)] | - |
| *Leucothrix mucor* | 2/4 | 1/4 | - | - | - | Yes, thiosulfate oxidation [[37](#_ENREF_37)] |

**References**

1. Pandey A, Naik MM, Dubey SK. Organic metabolites produced by *Vibrio parahaemolyticus* strain An3 isolated from Goan mullet inhibit bacterial fish pathogens. Afr J Biotechnol. 2010; 9: 7134-7140.

2. Lambert C, Nicolas JL, Cilia V, Corre S. *Vibrio pectenicida* sp. nov., a pathogen of scallop (*Pecten maximus*) larvae. Int J Syst Bacteriol. 1998; 48: 481-487.

3. Curson ARJ, Fowler EK, Dickens S, Johnston AWB, Todd JD. Multiple DMSP lyases in the gamma-proteobacterium *Oceanimonas doudoroffii*. Biogeochemistry. 2012; 110: 109-119.

4. Pujalte M-J, Garay E. Proposal of *Vibrio mediterranei* sp. nov.: a new marine member of the genus *Vibrio*. Int J Syst Bacteriol. 1986; 36: 278-281.

5. Thompson F, Thompson C, Hoste B, Vandemeulebroecke K, Gullian M, Swings J. *Vibrio fortis* sp. nov. and *Vibrio hepatarius* sp. nov., isolated from aquatic animals and the marine environment. Int J Syst Evol Microbiol. 2003; 53: 1495-1501.

6. Haldar S, Chatterjee S, Sugimoto N, Das S, Chowdhury N, Hinenoya A, et al. Identification of *Vibrio campbellii* isolated from diseased farm-shrimps from south India and establishment of its pathogenic potential in an *Artemia* model. Microbiology. 2011; 157: 179-188.

7. Ben-Haim Y, Thompson F, Thompson C, Cnockaert M, Hoste B, Swings J, et al. *Vibrio coralliilyticus* sp. nov., a temperature-dependent pathogen of the coral *Pocillopora damicornis*. Int J Syst Evol Microbiol. 2003; 53: 309-315.

8. Garren M, Son K, Raina J-B, Rusconi R, Menolascina F, Shapiro OH, et al. A bacterial pathogen uses dimethylsulfoniopropionate as a cue to target heat-stressed corals. ISME J. 2014; 8: 999-1007.

9. Noguerola I, Blanch AR. Identification of *Vibrio* spp. with a set of dichotomous keys. J Appl Microbiol. 2008; 105: 175-185.

10. Lee JV, Donovan TJ, Furniss AL. Characterization, taxonomy, and emended description of *Vibrio metschnikovii*. Int J Syst Evol Microbiol. 1978; 28: 99-111.

11. Beleneva I, Kukhlevskii A. Characterization of *Vibrio gigantis* and *Vibrio pomeroyi* isolated from invertebrates of Peter the Great Bay, Sea of Japan. Microbiology. 2010; 79: 402-407.

12. Hashem M, El-Barbary M. *Vibrio harveyi* infection in Arabian Surgeon fish (*Acanthurus sohal*) of Red Sea at Hurghada, Egypt. Egypt J Aquat Res. 2013; 39: 199-203.

13. Gomez-Gil B, Thompson FL, Thompson CC, Swings J. *Vibrio pacinii* sp nov., from cultured aquatic organisms. Int J Syst Evol Microbiol. 2003; 53: 1569-1573.

14. Knoblauch C, Sahm K, Jorgensen BB. Psychrophilic sulfate-reducing bacteria isolated from permanently cold Arctic marine sediments: description of *Desulfofrigus oceanense* gen. nov., sp. nov., *Desulfofrigus fragile* sp. nov., *Desulfofaba gelida* gen. nov., sp. nov., *Desulfotalea psychrophila* gen. nov., sp. nov. and *Desulfotalea arctica* sp. nov. Int J Syst Evol Microbiol. 1999; 49: 1631-1643.

15. Shaaban M, Maskey RP, Wagner-Dobler I, Laatsch H. Pharacine, a natural p-cyclophane and other indole derivatives from *Cytophaga* sp. strain AM13. 1 1. J Nat Prod. 2002; 65: 1660-1663.

16. Janssen PH, Liesack W. Succinate decarboxylation by *Propionigenium maris* sp. nov., a new anaerobic bacterium from an estuarine sediment. Arch Microbiol. 1995; 164: 29-35.

17. Watson J, Matsui GY, Leaphart A, Wiegel J, Rainey FA, Lovell CR. Reductively debrominating strains of *Propionigenium maris* from burrows of bromophenol-producing marine infauna. Int J Syst Evol Microbiol. 2000; 50: 1035-1042.

18. Kohda C, Ando T, Nakai Y. Isolation and characterization of anaerobic indole and skatole degrading bacteria from composting animal wastes. J Gen Appl Microbiol. 1997; 43: 249-255.

19. Schink B, Zeikus JG. *Clostridium thermosulfurogenes* sp. nov., a new thermophile that produces elemental sulphur from thiosulphate. J Gen Microbiol. 1983; 129: 1149-1158.

20. Idris EE, Iglesias DJ, Talon M, Borriss R. Tryptophan dependent production of indole-3-acetic acid (IAA) affects level of plant growth promotion by *Bacillus amyloliquefaciens* FZB42. Mol Plant Microbe Interact. 2007; 20: 619-626.

21. Read TD, Peterson SN, Tourasse N, Baillie LW, Paulsen IT, Nelson KE, et al. The genome sequence of *Bacillus anthracis* Ames and comparison to closely related bacteria. Nature. 2003; 423: 81-86.

22. Sass A, Rutters H, Cypionka H, Sass H. *Desulfobulbus mediterraneus* sp. nov., a sulfate-reducing bacterium growing on mono-and disaccharides. Arch Microbiol. 2002; 177: 468-474.

23. Ahn Y-B, Kerkhof LJ, Haggblom MM. *Desulfoluna spongiiphila* sp. nov., a dehalogenating bacterium in the *Desulfobacteraceae* from the marine sponge *Aplysina aerophoba*. Int J Syst Evol Microbiol. 2009; 59: 2133-2139.

24. Suzuki D, Ueki A, Amaishi A, Ueki K. *Desulfoluna butyratoxydans* gen. nov., sp. nov., a novel Gram-negative, butyrate-oxidizing, sulfate-reducing bacterium isolated from an estuarine sediment in Japan. Int J Syst Evol Microbiol. 2008; 58: 826-832.

25. Appia-Ayme C, Little PJ, Matsumoto Y, Leech AP, Berks BC. Cytochrome complex essential for photosynthetic oxidation of both thiosulfate and sulfide in *Rhodovulum sulfidophilum*. J Bacteriol. 2001; 183: 6107-6118.

26. Pukall R, Buntefuss D, Fruhling A, Rohde M, Kroppenstedt RM, Burghardt J, et al. *Sulfitobacter mediterraneus* sp. nov., a new sulfite-oxidizing member of the α-Proteobacteria. Int J Syst Bacteriol 1999; 49: 513-519.

27. Reisch CR, Crabb WM, Gifford SM, Teng Q, Stoudemayer MJ, Moran MA, et al. Metabolism of dimethylsulphoniopropionate by *Ruegeria pomeroyi* DSS‐3. Mol Microbiol. 2013; 89: 774-791.

28. Johnson TL, Palenik B, Brahamsha B. Characterization of a functional vanadium‐dependent bromoperoxidase in the marine cyanobacterium *Synechococcus* sp. cc93111. J Phycol. 2011; 47: 792-801.

29. Inze D, Follin A, Van Lijsebettens M, Simoens C, Genetello C, Van Montagu M, et al. Genetic analysis of the individual T-DNA genes of *Agrobacterium tumefaciens;* further evidence that two genes are involved in indole-3-acetic acid synthesis. Mol Gen Genet. 1984; 194: 265-274.

30. Iizuka T, Jojima Y, Fudou R, Hiraishi A, Ahn JW, Yamanaka S. *Plesiocystis pacifica* gen. nov., sp. nov., a marine myxobacterium that contains dihydrogenated menaquinone, isolated from the Pacific coasts of Japan. Int J Syst Evol Microbiol. 2003; 53: 189-195.

31. Patten CL, Glick BR. Role of *Pseudomonas putida* indoleacetic acid in development of the host plant root system. Appl Environ Microbiol. 2002; 68: 3795-3801.

32. Itoh N, Morinaga N, Kouzai T. Purification and characterization of a novel metal-containing nonheme bromoperoxidase from *Pseudomonas putida*. Biochim Biophys Acta. 1994; 1207: 208-216.

33. Hur HG, Sadowsky MJ, Wackett LP. Metabolism of chlorofluorocarbons and polybrominated compounds by *Pseudomonas putida* G786 (pHG-2) via an engineered metabolic pathway. Appl Environ Microbiol. 1994; 60: 4148-4154.

34. Vermeij P, Kertesz MA. Pathways of Assimilative Sulfur Metabolism in *Pseudomonas putida*. J Bacteriol. 1999; 181: 5833-5837.

35. Yang LH, Xiong H, Lee OO, Qi SH, Qian PY. Effect of agitation on violacein production in *Pseudoalteromonas luteoviolacea* isolated from a marine sponge. Lett Appl Microbiol. 2007; 44: 625-630.

36. Jiang Z, Boyd KG, Mearns-Spragg A, Adams DR, Wright PC, Burgess JG. Two diketopiperazines and one halogenated phenol from cultures of the marine bacterium, *Pseudoalteromonas luteoviolacea*. Nat Prod Lett. 2000; 14: 435-440.

37. Grabovich MY, Muntyan MS, Lebedeva VY, Ustiyan VS, Dubinina GA. Lithoheterotrophic growth and electron transfer chain components of the filamentous gliding bacterium *Leucothrix mucor* DSM 2157 during oxidation of sulfur compounds. FEMS Microbiol Lett. 1999; 178: 155-161.
